# Some things never change: multi-decadal stability in humpback whale calling repertoire on Southeast Alaskan foraging grounds

**DOI:** 10.1038/s41598-018-31527-x

**Published:** 2018-09-27

**Authors:** Michelle E. H. Fournet, Christine M. Gabriele, David C. Culp, Fred Sharpe, David K. Mellinger, Holger Klinck

**Affiliations:** 1 Department of Fisheries and Wildlife, Oregon State University, Corvallis, Oregon, USA; 2 Alaska Whale Foundation, Petersburg, Alaska, USA; 3 Humpback Whale Monitoring Program, Glacier Bay National Park and Preserve, Gustavus, Alaska, USA; 4 Cooperative Institute for Marine Resources Studies, Oregon State University and NOAA Pacific Marine Environmental Laboratory, Newport, Oregon, USA; 5 Bioacoustics Research Program, Cornell Lab of Ornithology, Ithaca, USA

## Abstract

Investigating long term trends in acoustic communication is essential for understanding the role of sound in social species. Humpback whales are an acoustically plastic species known for producing rapidly-evolving song and a suite of non-song vocalizations (“calls”) containing some call types that exhibit short-term stability. By comparing the earliest known acoustic recordings of humpback whales in Southeast Alaska (from the 1970’s) with recordings collected in the 1990’s, 2000’s, and 2010’s, we investigated the long-term repertoire stability of calls on Southeast Alaskan foraging grounds. Of the sixteen previously described humpback whale call types produced in Southeast Alaska, twelve were detected in both 1976 and 2012, indicating stability over a 36-year time period; eight call types were present in all four decades and every call type was present in at least three decades. We conclude that the conservation of call types at this temporal scale is indicative of multi-generational persistence and confirms that acoustic communication in humpback whales is comprised of some highly stable call elements in strong contrast to ever-changing song.

## Introduction

Acoustic signaling can reveal key insights into animal behavior across a broad range of taxa. Acoustic signals are quantifiable, may be easily collected at little to no impact to the subject species, and can be directly compared across space and time^1,2^. Investigating long-term trends in acoustic communication is essential for understanding drivers of vocal change and the role of acoustic communication within a species’ life history^3,4^. This is particularly relevant for those species, including many vertebrates, whose acoustic repertoire is shaped by genetic, cultural, and functional processes and may shift within and between generations in response to changing ecological niches, environmental conditions, and human activities (e.g. black-capped chickadees (*Poecile atricapillus)*^5^, domestic fowl (*Gallus gallus)*^6^, harp seals (*Pagophilus groenlandicus*)^7^, killer whales (*Orcinus orca*)^8,9^, North Atlantic right whales (*Eubalaena glacialis*)^10,11^).

The degree of vocal stability and vocal change within an acoustic repertoire varies between taxa and species. The need to attract a mate, find food, or to avoid predators exerts strong selection pressure on systems of acoustic communication^12,13^. As a result, social structure is often reflected in vocal structure and calling behavior. African savanna elephants (*Loxodonta africana)*, for example, are far ranging socially complex animals, capable of maintaining relationships across great distances^14^. As a reflection, elephants produce low-frequency contact calls that can travel for several kilometers and contain identifying information^14–16^. The mating strategies of some wren species (family *Troglodytidae*) manifest in their song repertoires, with polygynous species exhibiting larger song repertoires than monogamous species, presumably due to female choice and increased male-male competition^17^. Highly social vervet monkeys (*Chlorocebus pygerythrus)* emit loud, discrete alarm calls to announce various predators^18^, a tactic that could be unnecessarily risky for an asocial species.

Social systems are reflected within acoustic communication for marine mammals as well^19^, although their natural history traits can make them more difficult to study. Assessing vocal change, or the lack thereof, is one method used to increase scientific understanding of call function and social structure as a whole^19^. For example, demonstrating temporal vocal stability in several cetacean species (*e*.*g*. sperm whales (*Physeter macrocephalus)*^3^, bottlenose dolphins (*Tursiops spp)*^20^, killer whales^9^) has resulted in a much richer understanding about conspecific interactions and the importance of consistent acoustic signals in maintaining social relationships in this taxon. Similarly, documenting temporal shifts in the vocal behavior of some marine mammals, most notably humpback whales (*Megaptera novaeangliae*) that are capable of rapid acoustic turnover^21^, has revealed a great deal about the importance of cultural exchange in mediating social interactions^22,23^.

Humpback whales are vocal, migratory baleen whales whose behaviors are temporally and geographically stratified between breeding and foraging grounds. While on low-latitude breeding grounds, male humpback whales produce song, a long, repetitive vocal display that is highly stereotyped over hours to days, but that progressively evolves over months to years, ultimately changing completely^24,25^. Males within a single breeding region in a given year usually conform to one song type, although in the Southern Hemisphere novel songs have been adopted in their entirety in as little as two years^21^. Unlike discrete imitation, in which a sound type is learned and then the acoustic properties of that sound stabilize, song “copying” in humpbacks is an iterative process seemingly without a fixed endpoint^26–28^. Song has been the focus of dedicated research for decades, and as a result acoustic characteristics that indicate vocal plasticity in this species are well-described^23,29–33^, but it is unknown whether humpback whales exhibit similar plasticity in their other vocalizations.

Humpback whales produce a suite of communication signals in addition to song known as “non-song vocalizations”^34–36^, or “social calls”^37,38^. These include any sound produced outside the patterned and repeated structure of song, but do not include the percussive non-vocal signals that result from breaching or flipper slapping^36,39,40^. Unlike the term song, which is widely accepted in the scientific literature across taxa, the use of the terms “non-song” and “social call” are not universal. In humpback whales some “non-song” vocalizations occur as song units^37,39^, and some “social calls” are produced by animals engaged in solitary behaviors and are thus not exclusively social^41^. In keeping with the broader body of animal communication literature, we therefore suggest and adopt the use of the term ‘call’ to describe vocalizations that occur independently of song^42–44^.

The degree to which calls form the basis of functional acoustic communication in humpback whales is only starting to be revealed^34,45,46^. Calls produced outside of a song context occur in both sexes, across all life stages, and can be heard consistently throughout the migratory range^34,36,40,47^. It is assumed that humpbacks worldwide produce calls; however, they have been formally described in only four populations^37–39,47–49^. Unlike song, calls may appear in pattered, non-stereotyped bout sequences or entirely in isolation from other vocalizations^35,37,48^. At times, calls appear as song units, indicating that their role may be multi-faceted^37^; at other times calls have been linked to foraging activities^41,50,51^ or social interactions^34,45^. While investigations into call use and function are increasing, scientific understanding of calling behavior remains limited.

One study to date has investigated the temporal stability of humpback whale calls. Rekdahl *et al*.^37^ identified 12 stable call types on an East Australian migratory corridor that were commonly produced and consistently detected over an 11 year time period^37^. This work demonstrated that some call types are conserved within a single generation of humpback whales, and set the framework for expanded studies into the conservation of call types in this species.

Using humpback whale calls recorded throughout Southeast Alaska during a 36-year period (1976 to 2012), we tested the hypothesis that the call type repertoire was stable across decades and assessed changes in fine-scale acoustic parameters of calls over time in this population. Between 1979 and 2012 the humpback whale population in Southeast Alaska increased from approximately 300 to at least 1,500 individuals^52,53^. On the North Pacific foraging grounds, site fidelity and reproduction – not immigration – are the principal drivers of population growth and genetic composition^54^. Age of first parturition in North Pacific humpback whales is 8–16 years^55,56^; thus, while the population was unlikely to have turned over completely in the duration of this study, by recording humpback whales over the 36-year duration of their recovery, we capture the acoustic behavior of both the original members of the population as well as subsequent generations. In doing so, this study seeks to describe how the vocal repertoire in this species is conserved across generations.

## Results

Within 114.9 hours of recordings, we identified a total of 914 high quality calls that fit our inclusion criteria. Recordings spanned 140 unique days within five separate years across four decades (Table 1). A total of 175 individuals were photographically identified in conjunction with this study in 1997, 2007 and 2008. The minimum number of whales present during recording periods ranged from 6 to 90 (Table 1). Animals were not localized in this study; it is unknown whether all whales within the area were contributing to the recorded calls, or which specific individuals were calling.

**Table 1:** Recording effort, number calls detected, and minimum number of individuals present for each data set; the number of individuals that were actively vocalizing is unknown.

Year	^#^of Days	^#^of Calls	^#^of Individuals Present
1976	4	279	6
1997	32	156	90
2007–2008	72	108	89
2012	32	371	26
Total	140	914	NA

### Classification

Sixteen known call types nested within for vocal classes were described in Southeast Alaska in 2012 (Table 2). A comprehensive description of each class and vocal type is available elsewhere^36^. Aural-visual analysis (AV) assigned 367 calls to the Low Frequency Harmonic (LFH) vocal class, 303 to the Pulsed (P) vocal class, 79 to the Noisy-Complex (NC) vocal class, and 165 to the Tonal (T) vocal class (Table 2).

**Table 2:** Number of calls detected from each vocal class – Low-Frequency-Harmonic (LFH), Noisy Complex (NC), Pulsed (P, and Tonal (T)– and call type for each decade. Sample sizes by vocal class are in bold.

Call Class	Call Type	N	1970’s	1990’s	2000’s	2010’s
LFH	Descending Moan	7	0	1	2	4
	Groan	16	8	3	2	3
	Growl	220	64	16	39	101
	Modulated Moan	10	5	2	0	3
	Variable Moan	8	0	3	2	3
	Whup	106	35	23	16	32
	All LFH	367	112	48	61	146
NC	Ahooga	27	0	20	2	5
	Ascending Shriek	19	3	8	3	5
	Descending Shriek	11	2	5	0	4
	Squeegie	12	0	2	1	9
	Trumpet	10	5	1	0	5
	All NC	79	10	35	6	28
P	Droplet	74	20	21	23	10
	Horse	10	3	3	0	4
	Swop	124	41	21	6	56
	Teepee	95	63	11	9	12
	All P	303	127	56	38	82
T	Feeding (All T)	165	30	17	3	115
Total	All	914	279	156	108	371

Rotated principal component analysis (PCA) output indicated that the use of two principal components was adequate to encompass the variability of the data (χ^2^ = 1409.98, p < 0.000001). The first rotated component (PC1) corresponded most closely to frequency parameters (lower, median, peak, and start frequency values; see Table 3 for variable descriptions), indicating that as PC1 increases calls generally increase in frequency. The second rotated component (PC2) corresponded most closely to temporal parameters (duration (negative relationship), amplitude modulation rate, frequency modulation rate, (Table 3)), indicating that as PC2 increases calls generally grow shorter and change more quickly.

**Table 3:** Acoustic parameters included in the classification and regression tree and principal component analyses.

Noise-Resistant Feature Set*	
Duration (s)*	Length of feature box
Bout	Number of repetitions of the same call type
Lower Frequency (Hz)*	Lower frequency limit of feature box
Start Frequency (Hz)	Starting frequency of fundamental
End Frequency (Hz)	Ending frequency of fundamental
Peak Frequency (Hz)	Frequency of the spectral peak
Median Frequency (Hz)*	Frequency where cumulative sum of cell values reach 50% of the total energy
Amplitude Modulation Rate*	Dominant rate of amplitude modulation
Frequency Modulation Rate*	Dominant rate of frequency modulation
Upsweep Fraction*	Fraction of time in which median frequency in one block is greater than that in preceding block, weighted by total energy in each block
Frequency Trend	Start F_0_/End F_0_
Aggregate Entropy*	A measure of total disorder in the call

Calls marked with a * are from the Noise-Resistant Feature Set (NRSF); all other parameters were extracted in Raven Pro 1.5. Parameters in bold – the ones involving frequency – were log-transformed to approximate the mammalian perception of pitch.

**Figure 1:**
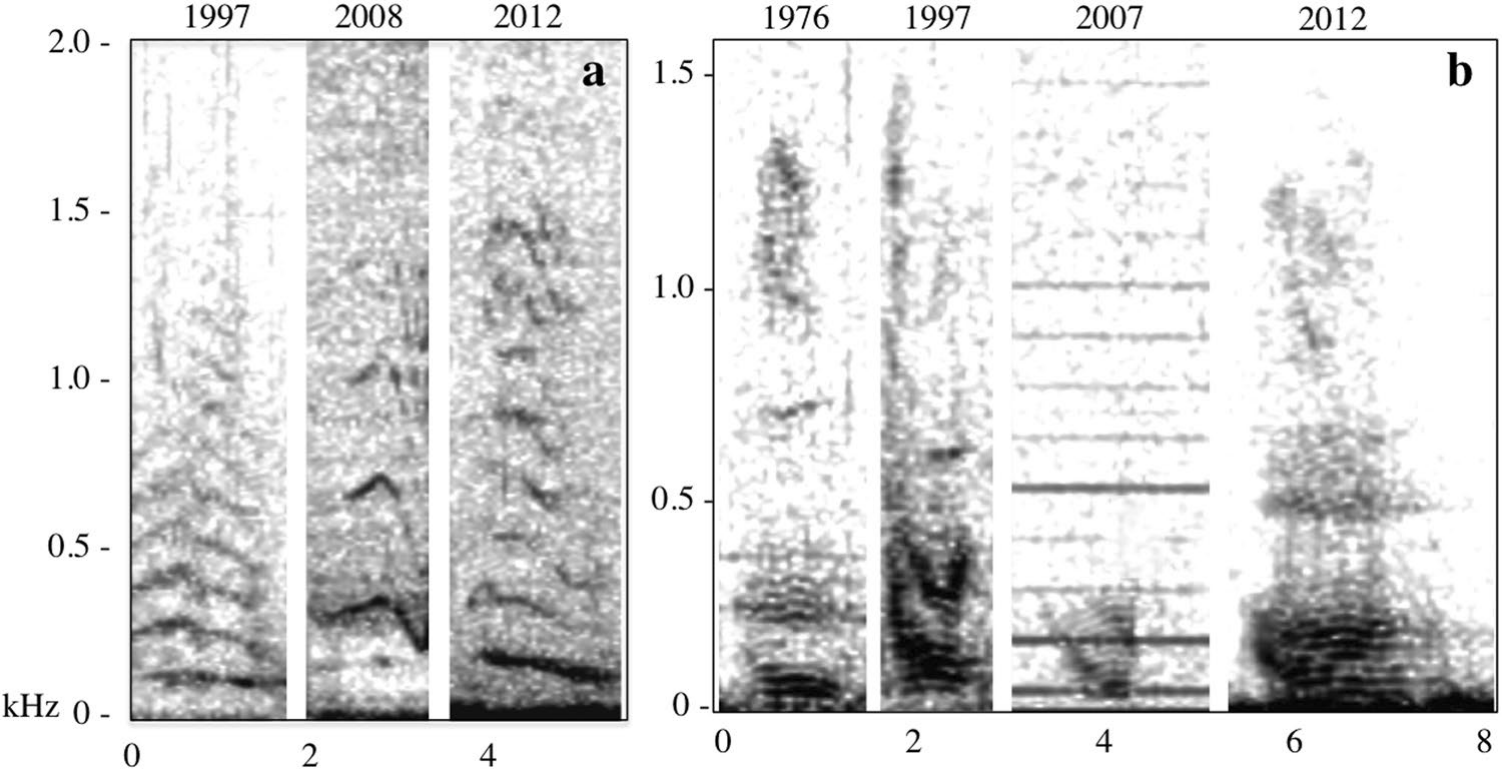
Spectrograms of the (a) descending moan call and (b) growl call over time (FFT 1375, 50% overlap, Hann window). Descending moan calls (n = 3) were commonly misclassified as growl calls (n = 220) despite structural differences discernable by a human observer.

A Classification and Regression Tree (CART) assigned 85% (root-node error, n = 754) of vocalizations to the same call type as AV classification (Table 4). Bout, duration, frequency ratio, PC1 and amplitude modulation were all important splitting variables. A random forest analysis correctly classified most of the calls (out-of-bag error rate (OOB) = 27%). Consistent with the CART analysis, the variables most important for splitting decisions were amplitude modulation, duration, bout, median frequency, PC1 and frequency ratio. Misclassifications were common among call types with low sample sizes^36^. These calls were re-reviewed manually by a second observer; upon observer agreement calls were categorized according to AV classification (Fig. 1).

**Table 4:** Confusion matrix of classification and regression tree (CART) output (top) compared to Aural-Visual (AV) call type assignment (left). Overall classification agreement was equal to 85%.

Call Type	n=	Ahooga	Asc. Shreik	Desc. Moan	Desc. Shriek	Droplet	Feed	Groan	Growl	Horse	Mod. Moan	Squeegie	Swop	Teepee	Trumpet	Var. Moan	Whup	
Ahooga	27	**25**	0	0	0	0	0	0	0	0	0	0	0	0	2	0	0	93%
Asc. Shriek	19	0	**17**	0	0	0	0	0	0	0	0	0	1	0	1	0	0	89%
Desc. Moan	7	0	0	**3**	0	0	0	0	3	0	0	0	0	0	0	0	1	43%
Desc. Shriek	11	1	2	0	**0**	0	3	0	2	0	0	0	1	0	1	0	1	0%
Droplet	74	0	0	0	0	**53**	0	1	1	0	0	2	10	2	0	0	5	72%
Feed	165	1	0	0	0	0	**160**	0	3	0	0	0	1	0	0	0	0	97%
Groan	16	0	0	1	0	0	0	**12**	2	0	0	0	0	0	0	0	1	75%
Growl	220	0	0	0	0	0	1	0	**200**	0	0	0	3	3	0	0	13	91%
Horse	10	0	1	0	0	0	0	2	0	**3**	0	2	1	1	0	0	0	30%
Mod. Moan	10	0	0	1	0	0	0	1	1	0	**6**	0	1	0	0	0	0	60%
Squeegie	12	1	1	1	0	0	0	1	0	1	0	**4**	3	0	0	0	0	33%
Swop	124	0	1	0	0	3	0	0	1	1	1	0	**107**	4	2	0	4	86%
Teepee	95	0	0	0	0	2	0	0	4	0	0	0	6	**82**	0	0	1	86%
Trumpet	10	0	0	0	0	0	0	0	0	0	0	0	2	0	**6**	0	2	60%
Var. Moan	8	1	0	2	0	0	0	0	3	0	0	0	0	0	2	**0**	0	0%
Whup	106	0	0	0	0	1	0	0	23	0	1	0	5	0	0	0	**76**	72%

### Temporal Stability

**Figure 2:**
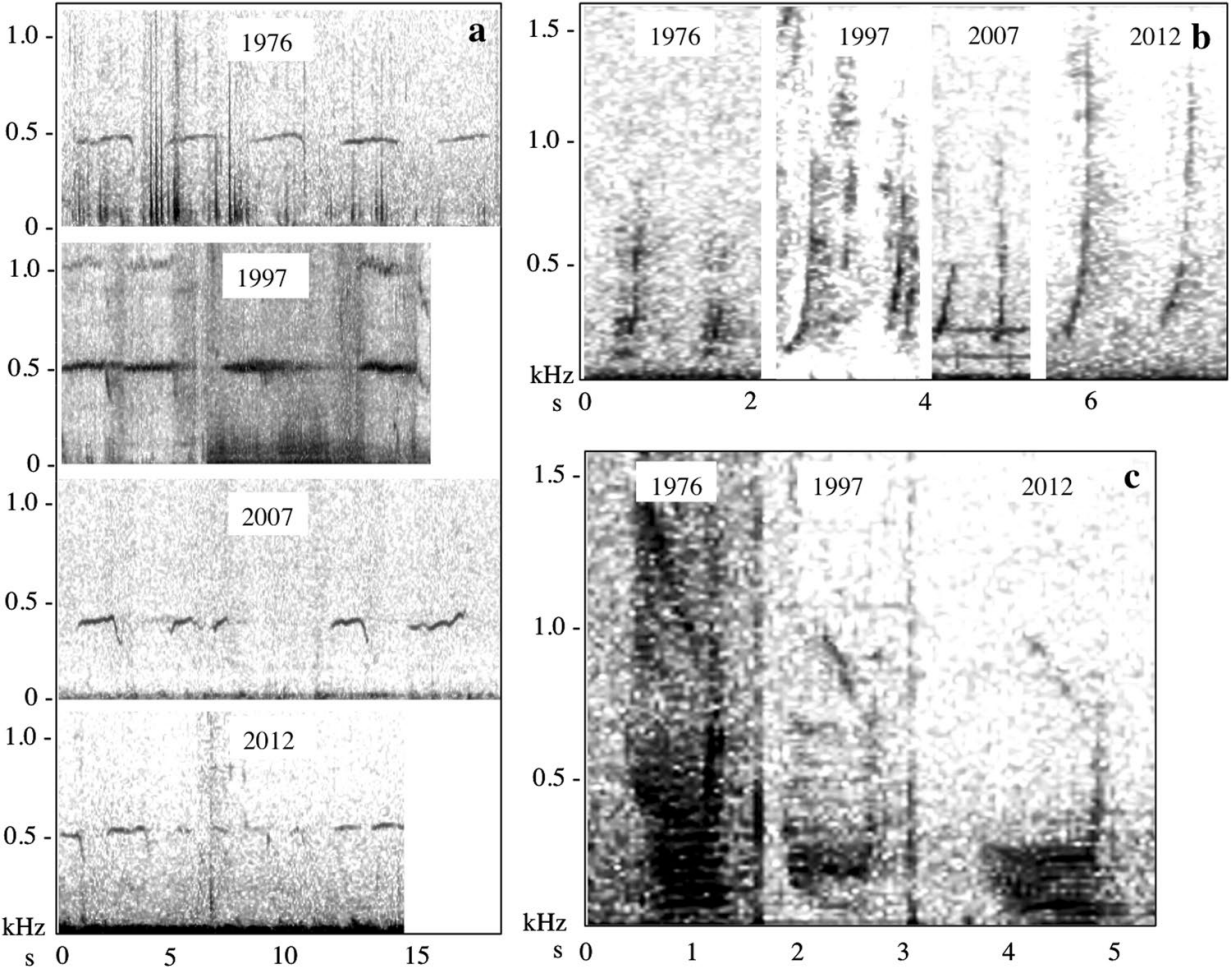
Spectrograms of commonly produced call types found in Southeast Alaska over time. (a) Feeding call examples by year (FFT 4771, 50% overlap, Hann window). (b) Droplet call examples by year (FFT 1375, 50% overlap, Hann window). (c) Whup call examples by year (FFT 1375, 50% overlap, Hann window).

**Figure 3:**
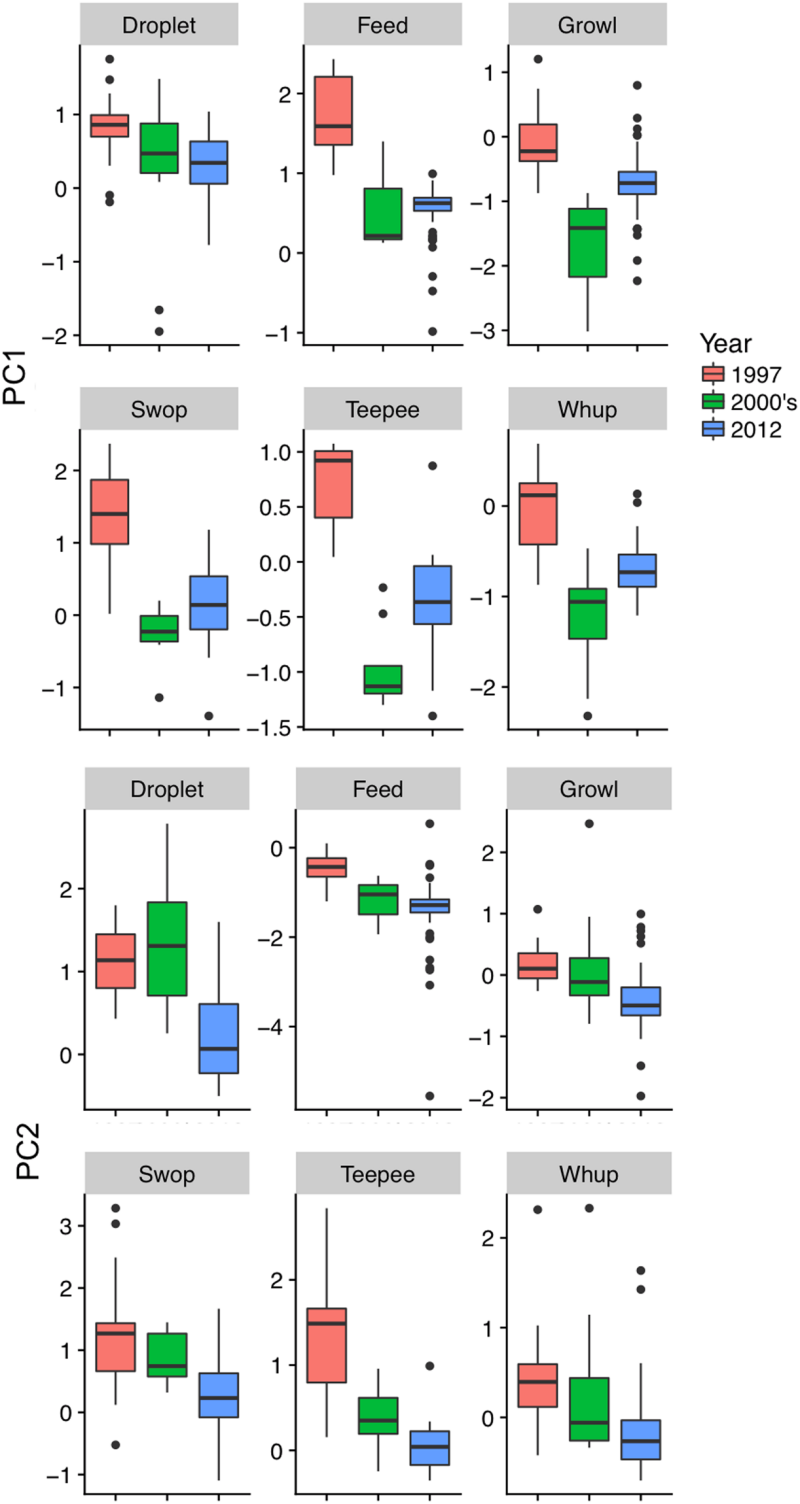
Boxplots of rotated principal component values for a selection of stable call types across time. PC1 corresponds to frequency parameters. PC2 corresponds to temporal parameters.

Twelve of the sixteen call types were found in both 1976 and 2012 (Table 2, Fig. 1, Fig. 2), and all call types were detected in at least three decades. Eight call types were represented in all four decades. Initial data analysis indicated that there were no significant differences in acoustic parameters between 2007 and 2008 (*χ*^2^ > 4, p > 0.1) and those years were pooled to ensure a large enough sample size for statistical inference across call types and between decades. The acoustic parameters of calls in this study were variable, though within the margin of variability reported for each call type (Table 5)^36^, and in some cases fine-scale acoustic parameters varied significantly between decades (Table 6, Fig. 3).

**Table 5:** Means (bold) and standard deviations of selected acoustic parameters for the subset of call types found across all four decades.

											PC-1		PC-2	
Type		Variable	1970’s		1990’s		2000’s		2010’s		*χ* ^2^	P	*χ* ^2^	P
Low-Frequency Harmonic	Groan	**N**	**8**		**3**		**2**		**3**		**4.69**	**0.095**	**1.0**	**0.6**
		**Low Freq (Hz)**	**53.4**	−33.8	**85.3**	−60.5	**55.7**	−11.2	**165.1**	−67.2				
		**Peak Freq (Hz)**	**152.1**	−116.7	**254.8**	−136.8	**119.8**	−9.5	**226.1**	−106				
		**Duration (s)**	**2.5**	−0.9	**8.5**	−8.1	**3.1**	−1.7	**3.3**	−1.6				
	Growl	**N**	**64**		**16**		**39**		**101**		**78.6**	**>*0***.***001***	**45.00**	**>*0***.***001***
		**Low Freq (Hz)**	**47.6**	−28.9	**64**	−40.3	**33.7**	−16.8	**57.8**	−35.4				
		**Peak Freq (Hz)**	**149.6**	−41.4	**253.7**	−189.2	**78.4**	−35.8	**104**	−64.9				
		**Duration (s)**	**0.8**	−0.3	**0.8**	−0.2	**0.9**	−0.4	**1**	−0.7				
	Whup	**N**	**35**		**23**		**16**		**32**		**37.34**	**>*0***.***001***	**17.61**	**>*0***.***001***
		**Low Freq (Hz)**	**60.5**	−26.5	**67.1**	−39	**31.5**	−9.6	**53.6**	−23				
		**Peak Freq (Hz)**	**176.4**	−87.8	**199.4**	−103.3	**85.8**	−37.8	**93.4**	−45.3				
		**Duration (s)**	**0.8**	−0.3	**0.7**	−0.2	**0.6**	−0.3	**0.7**	−0.2				
Noisy Complex	Ascending Shriek	**N**	**3**		**8**		**3**		**5**		**6.5**	***0***.***038***	**11.44**	***0***.***003***
		**Low Freq (Hz)**	**812.9**	−478.6	**1019.5**	−717	**1749**	−92.8	**1327.7**	−1115				
		**Peak Freq (Hz)**	**1293.8**	−702.9	**1359.3**	−651.8	**2110.3**	−195.8	**1899.2**	−1124.8				
		**Duration (s)**	**1.6**	−0.6	**1.4**	−0.3	**1.5**	−0.8	**1.6**	−0.7				
Pulsed	Droplet	**N**	**20**		**21**		**23**		**10**		**7.04**	**>*0***.***03***	**13.99**	**>*0***.***001***
		**Low Freq (Hz)**	**72.8**	−34	**126.9**	−80	**161.1**	−97.2	**164.5**	−72.3				
		**Peak Freq (Hz)**	**199.7**	−119.4	**293.3**	−170.5	**272.7**	−99.5	**311.2**	−165				
		**Duration (s)**	**0.4**	−0.2	**0.4**	−0.2	**0.3**	−0.2	**0.6**	−0.4				
	Swop	**N**	**41**		**21**		**6**		**56**		**36.68**	**>*0***.***001***	**20.99**	***0***.***002***
		**Low Freq (Hz)**	**87.9**	−70.1	**190.9**	−105.8	**75**	−38.1	**139.8**	−113.2				
		**Peak Freq (Hz)**	**309.9**	−203.5	**665**	−547.8	**191.6**	−50.9	**270.1**	−237.4				
		**Duration (s)**	**0.3**	−0.2	**0.7**	−0.6	**0.5**	−0.4	**0.6**	−0.3				
	Teepee	**N**	**63**		**11**		**9**		**12**		**21.51**	**>*0***.***001***	**16.04**	**>0**.**001**
		**Low Freq (Hz)**	**80.3**	−49	**111.2**	−43	**54.9**	−12.9	**76.3**	−40				
		**Peak Freq (Hz)**	**180.5**	−96.5	**339.6**	−88	**126.5**	−61.8	**131**	−109.6				
		**Duration (s)**	**0.4**	−0.2	**0.3**	−0.1	**0.5**	−0.4	**0.6**	−0.2				
Tonal	Feed	**N**	**10**		**17**		**3**		**45**		**44.09**	**>*0***.***001***	**37.05**	**>*0***.***001***
		**Low Freq (Hz)**	**424.8**	−30.6	**517.1**	−221.8	**329.1**	−226	**430.7**	−114.6				
		**Peak Freq (Hz)**	**496.9**	−34.6	**630.8**	−237.5	**426.2**	−148.9	**476.8**	−80.7				
		**Duration (s)**	**1.8**	−0.6	**3.3**	−1.7	**2.4**	−2.7	**3.6**	−2.8				

PC1 corresponds to frequency components. PC2 corresponds to temporal components”. Chi-squared statistic and *P*-values refer to results from Kruskal-Wallis test for equality of medians. See Table 6 for detailed results of Dunn’s test for multiple comparisons. Significant differences are italicized and underlined.

**Table 6:** *P*-values from Dunn’s test pairwise comparisons for differences in median rotated principal components between decades.

Type	Variable		1997	2007–2008
Droplet	PC1	2007–2008	0.1890	—
		2012	*0*.*0380*	0.8900
	PC2	2007–2008	1.0000	—
		2012	*0*.*0051*	*0*.*0008*
Swops	PC1	2007–2008	*0*.*0000*	—
		2012	*0*.*0000*	0.3400
	PC2	2007–2008	1.0000	—
		2012	*0*.*0000*	0.0850
Teepee	PC1	2007–2008	*0*.*0000*	—
		2012	*0*.*0051*	0.3124
	PC2	2007–2008	0.0620	—
		2012	*0*.*0002*	0.4755
Feed	PC1	2007–2008	*0*.*0030*	—
		2012	*0*.*0000*	1.0000
	PC2	2007–2008	0.2500	—
		2012	*0*.*0000*	1.0000
Growl	PC1	2007–2008	*0*.*0000*	—
		2012	*0*.*0013*	*0*.*0000*
	PC2	2007–2008	0.5300	—
		2012	*0*.*0000*	*0*.*0000*
Whup	PC1	2007–2008	*0*.*0000*	—
		2012	*0*.*0003*	*0*.*0116*
	PC2	2007–2008	0.3500	—
		2012	*0*.*0000*	0.1200

PC1 corresponds to frequency components. PC2 corresponds to temporal components. Significant differences between decades are italicized and underlined.

For all call types except for droplets, mean PC1 values were significantly higher in calls produced in 1997 (Fig. 3) than in either the 2007–08 or 2012 data sets. Mean PC1 values for droplets were highest in 1997, but mean PC1 values for droplets in 1997 were not significantly different than calls produced in the 2000’s. In general, calls produced in 1997 were higher in frequency than in the other two decades. PC1 values tended to be lowest for calls produced in 2007–08 and intermediate in 2012 (Fig. 3). There was no obvious linear change in PC1 values over time. Where significant differences occurred, PC2 values showed a general downward trend between 1997 and 2012 (Table 5); PC2 estimates indicate that across call types calls produced in 1997 were shorter and had higher modulation rates, and calls from 2012 were longer and had slower modulation rates (Fig. 3).

## Discussion

The results of this study indicate that some humpback whale non-song call types in Southeast Alaska are conserved in the acoustic repertoire at the decadal timescale. Twelve of the 16 call types were present in both the 1976 and 2012, demonstrating the persistence of these calls over 36 years. All other call types persisted over multiple decades between the 1997 and 2012. The absence of identical call types in recordings across the four decades does not necessarily indicate that certain call types were absent from the repertoire, since it is unlikely that the non-song calls aggregated for this study comprise the entire vocal repertoire of the entire population at any given time. Limited sampling effort (Table 1) might favor the recording of some call types over others; nonetheless, within the scope of this dataset the overall pattern of long-term conservation of call types within the acoustic repertoire is clear.

Demographic data are consistent with the assertion that stability at this temporal scale indicates call types are conserved across several generations. Given that females mature by the age of 13 and typically reproduce every 1–3 years^55–57^, new whales in our survey regions were born, reached sexual maturity, and gave birth to offspring that subsequently grew to sexual maturity and also gave birth over the 36-year duration of this study^58^. This is reflected in the dramatic increase in population size from the 1970’s to the 2010’s, which was estimated in 2008 to be more than five times as large as in 1979^52,53^. Demographic studies show that population growth in Southeast Alaska is primarily due to long-term maternally directed site fidelity and birth^54,55,59,60^ with little evidence of immigration into the feeding areas of the population^59^. In further support of this point, in Glacier Bay National Park, where humpback whale monitoring efforts have been underway since the 1970’s, almost half of the humpback whales first identified as calves returned to their maternal foraging grounds^55^. Thus, it is quite likely that throughout the study period multiple generations overlapped spatially and temporally within the survey region and were recorded by our hydrophones.

In this study, some acoustic parameters varied significantly, though non-linearly, across decades. Most vertebrate sounds demonstrate within-call variation, related to individual anatomy, behavioral or environmental context (see Fig. 2 for a visual example from this study), which does not contradict placement into call classes or types^19,61^. In the eastern Australian humpback whale population call types classified with high degrees of confidence also exhibit fine-scale acoustic variability over time^37^. In the same population, acoustic parameters of calls also varied with social context, though call type assignment was robust^48^. Similarly, Deecke *et al*.^61^ demonstrated changes in acoustic parameters of killer whale calls, although the overarching call structure remained stable^61^. Thus, although there were observed differences in acoustic parameters between decades, the persistence of the call types themselves in the acoustic repertoire should be considered stable.

There were some prominent trends in fine-scale acoustic behavior between years. Calls from 1997 were consistently higher and shorter than other time periods and calls of almost all types grew longer from 1997 to 2012. We offer a few possible explanations. Individuals may have adjusted vocal parameters in response to ambient noise conditions; to avoid acoustic masking individuals must either increase their calling amplitude or spectrally or temporally shift their vocalizations^62^. Alternatively, and equally plausibly, changes in acoustic parameters may reflect differences in social context rather than change over time. According to motivational-structural rules, mammalian vocal sounds encode information about a sender’s motivational state, enabling a receiver to assess the likelihood of certain behaviors occurring^63^. It has been suggested that motivational state is encoded in humpback whale calls on migratory corridors^46^. In eastern Australia humpback whales used call types that were higher in frequency at times associated with increased arousal levels (i.e. affiliating groups for mid-level arousal, groups of competing males for high arousal)^46,48^. In the present study, recordings made in 1997 were typically made in association with large groups of whales engaged in coordinated foraging events. In contrast, despite dedicated observer effort coordinated foraging was not observed in 2007 or 2008^64,65^, and whales in 2012 were typically foraging alone, in low densities, and in some cases were vocalizing in isolation^41,48^. Coordinated foraging is likely to increase arousal and necessitates fine-scale cooperative interactions between individuals^66^. This indicates a difference in audience and plausibly a difference in motivational state between datasets in the different decades. High-frequency, short-duration vocalizations that are frequency-modulated are thought to have an ‘appeasing effect’ on receivers^63^ and have been associated with groups of affiliating humpback whales^46^. Consistent with this theory, calls from 1997, where groups of animals were engaged in complex coordinated activities, were generally higher, shorter, and more frequency modulated than in decades where social interaction was more limited during recording periods.

Our result, that calls grew generally longer over the duration of this study, corroborates findings in other mysticetes, where call duration increased over time, possibly in response to elevated ambient noise^33,58,67^. However, statistically significant temporal differences in call features may or may not have practical significance. For example, PC2 values, which related to temporal features, were lower for droplet and teepee calls produced in the 2012 than in the 2000’s, indicating that calls in 2012 were longer in duration; the differences in mean duration, however, were only a fraction of a second. Because these calls are pulsed and often occur in short bouts^36,68^, a difference in a fraction of a second may reflect a longer duration between call units, or could be an artifact of temporal smearing with distance from the hydrophone. While every effort was made to account for differences in environmental conditions and recording units, the temporal parameters of a call can still be affected by attenuation, reverberation, and other propagation effects. Should these very fine-scale measurements be indicative of true variation in temporal characteristics, they still may not represent enough biological variability to be detectable, or meaningful to a receiver. Additional research into auditory discrimination in humpback whales would be extremely valuable.

In the absence of calibrated ambient sound recordings, demographic information, and fine-scale behavioral sampling, it is not possible to quantify whether motivation, ambient noise conditions, or individual variability contributed most to changes in calling behavior. Future investigation into acoustic variability as a function of social context and ambient noise on feeding grounds would be useful for testing hypotheses about acoustic communication in foraging humpback whales.

The selective pressures that maintain and shape characteristics of communication signals are dictated by social structure^13,69^. Species like killer whales that live in discrete family units with little or no natal dispersal exhibit temporally stable and stereotyped pod-specific vocalizations^70–72^. Bottlenose dolphin societies characterized as ‘fission-fusion’, with social affiliations ranging from ephemeral to long term, employ stable signature whistles to convey individual identity^73,74^. We have demonstrated that humpback whales also produce call types that persist across decadal time scales. Given the evidence of long-term affiliation between humpback whales at high latitudes^57,75,76^ we suggest that some call types may be used communicate identity over time and space. Acoustic identity cues are common across taxa (*e*.*g*. northern fur seal (*Callorhinus ursinus*) mother-pup recognition calls^77^, king penguin (*Aptenodytes patagonicus*) contact calls^78^, Mexican free-tailed bats (*Tadarida brasiliensis mexicana)* isolation calls^79^; see Tibbetts & Dale, 2007 for a review^80^), and – as evidenced by savanna elephants – can be valuable among far ranging social animals^15,16^. Some of the most commonly documented call types identified in this study, whups and growls, have been frequently identified elsewhere^38,39,49^, and may act as contact calls^34,45^. These call types are acoustically variable, and highly persistent over time on both migratory corridors and foraging grounds; this variability may reflect signature information used to confer identity over time. We propose that these call types are good starting points for investigating individual variation and recognition in this species.

Calls may also persist in the vocal repertoire in Southeast Alaska because they are functionally specific to foraging activities in this region. Feeding calls are behaviorally linked to foraging on Pacific herring (*Clupea pallasii*)^41,51,60^ and are closely matched to hearing capabilities of this prey, which are most sensitive in the 200–500 Hz range^81^. Under experimental conditions, playbacks of feeding calls have elicited a “flee and clump” response in Pacific herring, which presumably increases the whales’ foraging efficiency^82^. Feeding calls are most commonly documented among groups of whales, where they may coordinate individuals; however, they are also produced by solitary animals, and may serve a prey manipulation function in this context^41^. This would explain both the call stereotypy – feeding calls were correctly classified 98% of the time in this study (Fig. 2, Table 4) – and also differences in call parameters between decades, which are likely related to social context (*e*.*g*. solitary foragers versus group foragers) or prey behavior (*e*.*g*. school size, school location). Although this call type does not exclusively serve a social function, it is likely to persist within the humpback whale repertoire because it is closely coupled with prey biology and provides a direct benefit to the individual producing it.

Lastly, humpback whale calling behavior in the feeding grounds stands in stark contrast to singing behavior. The composition and structure of song changes within the breeding season resulting in progressive seasonal and inter-annual change^21,22,28^, whereas this study and the work of Rekdahl *et al*.^37^ using 11 years of audio data from the east Australia migratory corridor, make it clear that portions of the call repertoire persist with time^37^. Importantly, in the east Australian population stable call types were not used as song units in adjacent years^37^, implying – similar to this study where song was not documented concurrently with calls – that calls function independently of song. Finding this commonality across ocean basins and in contrasting portions of humpback whale migratory range is quite telling. We propose that conservation of call types may be as important to call function as novelty is to song function and encourage future investigations into humpback whale calling behavior to include this hypothesis.

## Conclusion

This study provides the first evidence that humpback whale call types persist across multiple generations. The longevity of calls from humpback whale feeding areas stands in marked contrast to the ever-changing humpback whale breeding-season song. Further investigation is needed to better understand the role of temporally stable calls as song units, as well as individual variation and call use across age and sex classes, social context, noise conditions, and between humpback whale populations.

## Methods

**Figure 4:**
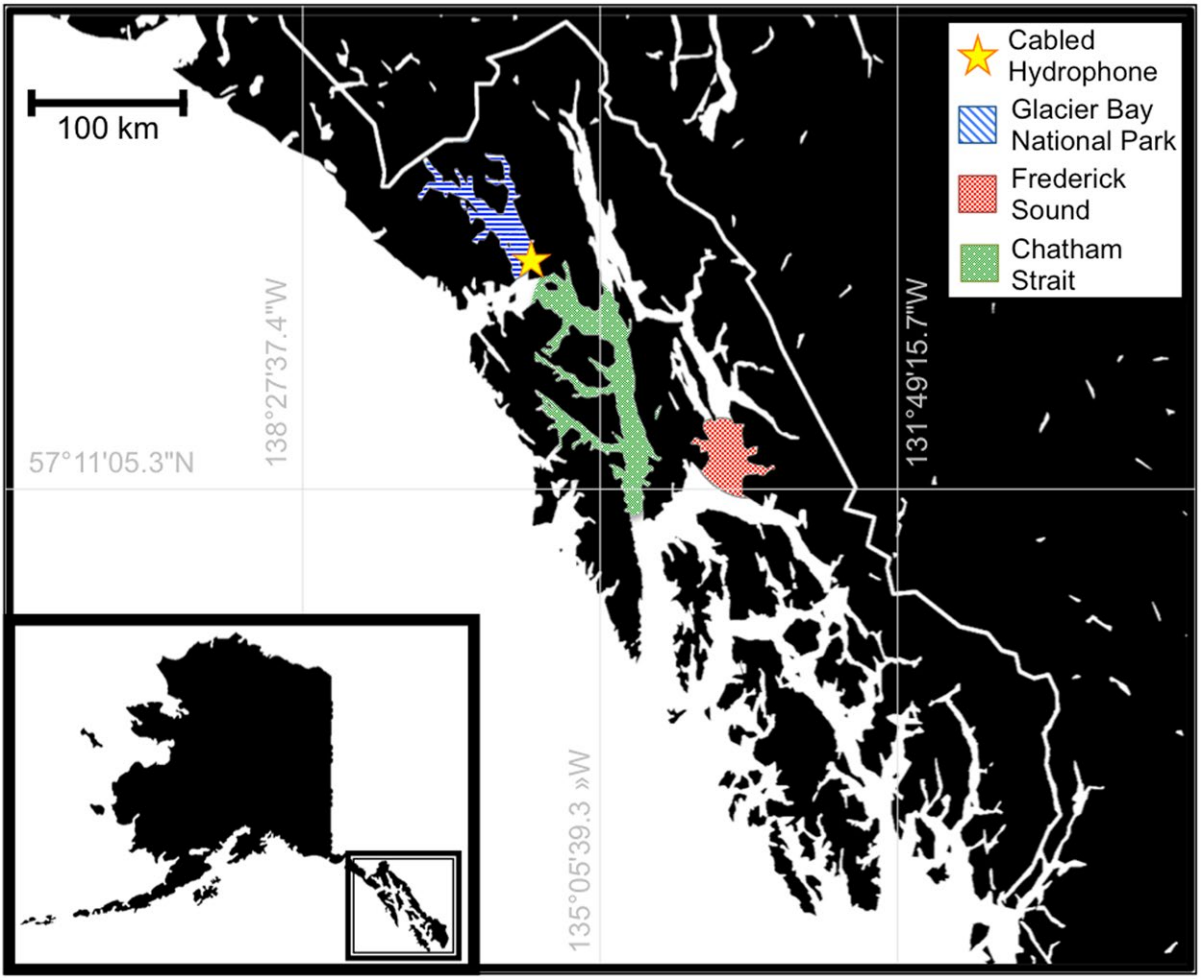
Map of survey areas. Blue denotes Glacier Bay National Park and Preserve, where recordings were made in 2007; the moored hydrophone is marked with a yellow star. Green denotes Chatham Strait, where recordings were made in 1997 Red denotes a portion of Frederick Sound, where recordings were made in 1976 and in 2012. (Map data ©2016 Google, adapted in Microsoft ® PowerPoint ® for Mac 2011).

We compiled acoustic recordings and associated whale sighting data from four data sets spanning 1976–2012. Acoustic data were collected using passive acoustic recording devices during summer months (June-August) on humpback whale foraging grounds throughout Southeast Alaska (Fig. 4, see supplementary material for recording equipment specifications) and paired with various forms of sighting data collection in the same locations.

Acoustic recordings from 1976 were made in Frederick Sound, Southeast Alaska (Fig. 4), were continuous during each individual recording, and were of variable length – ranging from thirty-two minutes to ninety-four minutes – and included behavioral narration on a separate recording track. Narration was summarized to glean the number of individuals present during recording. Acoustic recordings from 1997 were made in Frederick Sound and Chatham Strait as part of an ongoing investigation into coordinated group feeding in humpback whales. When possible, individuals were photographed concurrently with acoustic recordings; photographs were compared to the collaborative Southeast Alaska Humpback Whale Catalog^83^ for identification. A single experienced observer (FS) pre-processed these recordings prior to the inception of this study: Visually and aurally distinguishable calls were extracted from continuous recordings and were saved individually as sound “clips” with a 0.5 second buffer at the start and end of each call. Acoustic recordings from 2007 and 2008 were collected from a cabled hydrophone in Bartlett Cove, Glacier Bay National Park (Fig. 4) with a 30-seconds-per-hour recording cycle^45^. These data were reviewed by U.S. Navy acousticians to characterize the content of each sound sample. We analyzed only the samples annotated to contain humpback whale calls. Because these data are from a remote monitoring system and not a dedicated effort to record whales, the whales were often further from the hydrophone and as such, calls with high signal-noise-ratios (SNR) were rarer in 2007 and 2008 than in other years. For this reason acoustic samples from these years were pooled for analysis. Recordings from Glacier Bay were paired with photo-identification data collected in the directly adjacent regions as part of a long-term monitoring program^55^. Acoustic recordings from 2012 in Frederick Sound were collected in approximately 30 minute increments^36^; recordings from 2012 were made in conjunction with shore-based counts conducted from the 18.3 meter-tall Five Finger Lighthouse, Frederick Sound^41^.

Acoustic samples from the 1976 were recorded on four consecutive days and represent a limited number of individuals. These samples were not assumed to be an adequate representation of humpback whale vocal behavior at that time. Thus, while all acoustic samples were classified to assess repertoire stability over time, acoustic samples from 1976 were omitted from comparative statistical analyses of acoustic parameters.

Recordings from 1976, 2007, 2008, and 2012 were sampled at 44.1 kHz. Recordings from 1997 were originally recorded with a sampling rate of 22.05 kHz and were resampled at a rate of 44.1 kHz for consistency with other years. A 10 kHz low-pass filter was applied to all recordings; in the case of data from the 1990’s, the filter was applied both before and after resampling. We used a 10-pole Butterworth filter with corner frequency of 10 kHz. Because the frequency band of interest was below 10 kHz, up-sampling data from the 1990’s to standardize data for equivalent analysis did not result in the interpolation of data beyond the original recording range.

Spectrograms of acoustic recordings were created with Raven Pro 1.5 (Cornell Lab of Ornithology, Ithaca, NY) using a 0.093 s window length (4096 samples; filter bandwidth 15.5 Hz), Hann window, frequency resolution uncertainty of +/−5.4 Hz, and 75% overlap, and were constrained to the 10 Hz to 3 kHz frequency range to facilitate analysis. Recordings were manually reviewed in their entirety by a single experienced observer (MF). Calls were visually and aurally identified within each recording and annotated in the time-frequency domain of the spectrogram. Recordings made in 1976, 2007, 2008, and 2012 were prepared to match the formatting of sound clips from 1997: acoustic samples containing calls were extracted from continuous files with a 0.5 s buffer adjacent to the calls’ start and end times. The SNR of each extracted sample was calculated using the method described by Mellinger and Bradbury^84^; to be considered for analysis, calls had to have visually distinguishable start and end points, be non-overlapping, and have a SNR of at least 10 dB above ambient noise levels^36,37,39^.

Differences in recording equipment and sampling protocol no doubt manifest in the acoustic data. In particular, because high frequency components attenuate more quickly than low frequency components, recordings made further away from the animals are likely to have lost energy in the upper ranges, and temporal patterns may be less evident. To adjust for this, high frequency acoustic parameters that may be sensitive to differences in recording equipment were not incorporated into quantitative classification analyses (*e*.*g*. high frequency, bandwidth). To further account for variation in recording equipment, salient acoustic features were extracted using the Noise-Resistant Feature Set (NRFS) measurement suite included in the MATLAB-based program Osprey^36,84^ (Table 3), with the change that the de-noising step was not performed before calculating the measurement values. The NRSF was designed for detection and classification of marine animal sounds across variable noise conditions. Rather than extracting measurements from an observer-drawn annotation box, the NRSF draws a smaller time-frequency region (“feature box”) in which the energy is ranked and summed within the sound relative to background noise. By doing this the loudest parts of the spectrogram have the strongest influence over the measured values, which allows for more standard measurements across recording conditions. Finer scale time-frequency measurements were made in Raven Pro 1.5 (Table 3). Fine-scale measurements were made on the fundamental frequency for harmonic sounds; for amplitude-modulated sounds containing a broadband component, measurements were made on the lowest-frequency component of the call^37,39^. To account for the mammalian perception of pitch, which is approximately logarithmic rather than linear^85^, frequency parameters were log-transformed^36,39^ (Table 3). The same time-frequency parameters were input into a Principal Component Analysis (PCA) in order to aggregate variables for classification and comparative analyses (*psych* package)^86^. A varimax rotation was applied to maximize loading and facilitate variable interpretation^39,60^.

Calls were classified aurally and visually (AV) into previously described vocal classes, and call types by a single experienced observer (MF) using the randomization method described by Fournet *et al*.^36^. Using the time-frequency parameters of Table 3 and rotated principal components, a non-parametric classification and regression tree (CART) with cross-validation was run in R on the aggregated dataset to assess the likelihood that calls were correctly classified by AV analysis (*rpart* package)^37,38,87^. A random forest method was then performed using the same acoustic parameters with (*randomForest* package)^88^. These two methods are emerging as the preferred method for classification of humpback whale calls as they are robust to non-normal datasets and outliers. Further, random forest analyses improve predictive accuracy by using a bootstrapping technique that determines the OOB error, or prediction uncertainty, associated with each classification tree, rather than just one. The number of predictors randomly selected at a node for splitting was set to three, and 1000 trees were grown^38,89^.

Non-song call types can be highly variable and in some cases appear to exist along a continuum^36,39,46,49^. In the event of low classifier agreement call types with very small sample sizes (>13) were manually re-reviewed by a second observer (DC); if observer agreement was consistent calls were grouped according to AV classification. Once calls were classified, the presence of call types was compared between years. To reduce subjectivity, only call types previously described for this population were included in analysis; as the goal of this paper was not to describe new call types samples that could not be classified to known types were omitted from analysis. For this reason it is possible that call types not analyzed in this study may exhibit long term stability, or lack therefor.

Within each vocal class a set of call types that exhibited stability across four decades and were found in large enough sample sizes were selected for fine-scale comparison of acoustic parameters over time. A Bartlett’s test with a significance level (α) of 0.05 indicated that, in almost all cases, the assumption of equal variance between decades was not met. To account for this and for non-normally distributed data, the non-parametric Kruskal-Wallis test was used to assess significant differences in median call parameters between decades (α = 0.05). We used a post-hoc Dunn’s test with a Bonferroni correction for all relevant pairwise comparisons, in the case of ties z-quantiles were used^90^. All analyses were conducted in R version 3.3.3^87^.

The dataset analyzed in the current study is available from the corresponding author on request. Acoustics samples of each call type can be found at mfournet.wordpress.com/sounds

## Electronic supplementary material

Supplementary Information

Sound File 1

Sound File 2

Sound File 3

Sound File 4
