# Supplementary material for: Some things never change: multi-decadal stability in humpback whale calling repertoire on Southeast Alaskan foraging grounds
Source: Sci Rep. 2018 Sep 27;8:13186. doi: 10.1038/s41598-018-31527-x (PMC6160409; doi:10.1038/s41598-018-31527-x)
Supplement: Supplementary file 1 — Supplementary Information [file 41598_2018_31527_MOESM1_ESM.docx]

**Some things never change: multi-decadal stability in humpback whale calling repertoire on Southeast Alaskan foraging grounds**

*Michelle E. H. Fournet^1,2^, Christine M. Gabriele^3^, David C. Culp^1^, Fred Sharpe^2^, David K. Mellinger^4,5^, Holger Klinck^6^

^1^ Department of Fisheries and Wildlife, Oregon State University

^2^ Alaska Whale Foundation

^3^ Humpback Whale Monitoring Program, Glacier Bay National Park and Preserve

^4^ Cooperative Institute for Marine Resources Studies, Oregon State University

^5^ NOAA Pacific Marine Environmental Laboratory

^6^ Bioacoustics Research Program, Cornell Lab of Ornithology

*Corresponding author can be reached at michelle.fournet@gmail.com

Supplementary Information- Acoustic sampling regimes including hydrophone specifications, recording cycles, and locations.

| **Year** | **1976** | **1997** | **2007 & 2008** | **2012** |
| --- | --- | --- | --- | --- |
| Hydrophone model | unknown | Offshore Acoustics #96b | ITC 8215A | Cetacean Research Technologies C-55 |
| Sampling Rate | 44.1 kHz | 22.05 kHz | 44.1 kHz | 44.1 kHz |
| Hydrophone Sensitivity | unavailable | -154 dB ±4dB re V/μPa at 6-14,000 Hz | -174 dB ±2dB re 1 V/μPa at 1-10,000 Hz | -165 dB ±3dB re 1 V/μPa at 10-10,000 Hz |
| Deployment Method | Dipping (20 m) | Dipping (10 m) | Bottom-mounted  (52 m) | Dipping (20 m) |
| Location | Frederick Sound | Frederick Sound, Chatham Strait | Glacier Bay | Frederick Sound |
| Recording Cycle | Non-standardized | Non-standardized | 30 seconds from every hour | Non-standardized |
| Data Format | Continuous | Extracted clips | 30 second recordings | ~30 minute recordings |
| Contributor | R. Payne | F. Sharpe | C. Gabriele | M. Fournet |
